# Should the diagnosis of COPD be based on a single spirometry test?

**DOI:** 10.1038/npjpcrm.2016.59

**Published:** 2016-09-29

**Authors:** Tjard R Schermer, Bas Robberts, Alan J Crockett, Bart P Thoonen, Annelies Lucas, Joke Grootens, Ivo J Smeele, Cindy Thamrin, Helen K Reddel

**Affiliations:** 1 Department of Primary and Community Care, Radboud University Medical Center, Nijmegen, The Netherlands; 2 Department of Chest Diseases, Canisius Wilhelmina Hospital, Nijmegen, The Netherlands; 3 School of Health Sciences, University of South Australia, Adelaide, SA, Australia; 4 Diagnostiek voor U, Eindhoven, The Netherlands; 5 Dutch College of General Practitioners, Utrecht, The Netherlands; 6 Woolcock Institute of Medical Research, University of Sydney, Sydney, NSW, Australia

## Abstract

Clinical guidelines indicate that a chronic obstructive pulmonary disease (COPD) diagnosis is made from a single spirometry test. However, long-term stability of diagnosis based on forced expiratory volume in 1 s over forced vital capacity (FEV_1_/FVC) ratio has not been reported. In primary care subjects at risk for COPD, we investigated shifts in diagnostic category (obstructed/non-obstructed). The data were from symptomatic 40+ years (ex-)smokers referred for diagnostic spirometry, with three spirometry tests, each 12±2 months apart. The obstruction was based on post-bronchodilator FEV_1_/FVC < lower limit of normal (LLN) and <0.70 (fixed ratio). A total of 2,352 subjects (54% male, post-bronchodilator FEV_1_ 76.5% predicted) were studied. By LLN definition, 32.2% were obstructed at baseline, but 32.2% of them were no longer obstructed at years 1 and/or 2. By fixed ratio, these figures were 46.6 and 23.8%, respectively. Overall, 14.3% of subjects changed diagnostic category by 1 year and 15.4% by 2 years when applying the LLN cut-off, and 15.1 and 14.6% by fixed ratio. Change from obstructed to non-obstructed was more likely for patients with higher body mass index (BMI) and baseline short-acting bronchodilator (SABA) users, and less likely for older subjects, those with lower FEV_1_% predicted, baseline inhaled steroid users, and current smokers or SABA users at year 1. Change from non-obstructed to obstructed was more likely for males, older subjects, current smokers and patients with lower baseline FEV_1_% predicted, and less likely for those with higher baseline BMI. Up to one-third of symptomatic (ex-)smokers with baseline obstruction on diagnostic spirometry had shifted to non-obstructed when routinely re-tested after 1 or 2 years. Given the implications for patients and health systems of a diagnosis of COPD, it should not be based on a single spirometry test.

## Introduction

Chronic obstructive pulmonary disease (COPD) is a prevalent chronic respiratory condition that is usually progressive and associated with an enhanced chronic inflammatory response in the airways and lungs to inhaled particles or gases.^1^ Current international clinical COPD guidelines state that spirometry is required to make the diagnosis.^1–4^ These guidelines state that the presence of a post-bronchodilator forced expiratory volume in 1 s (FEV_1_) over forced vital capacity (FVC) ratio below 0.70 confirms the presence of airway obstruction in subjects who are suspected of having COPD due to cumulative exposure to risk factors (e.g., tobacco smoke, occupational or indoor air pollution) and presence of respiratory symptoms that are compatible with COPD.^1–4^ There is increasing evidence that the use of an age- and sex-specific lower limit of normal value for FEV_1_/FVC would be a more appropriate approach when defining airway obstruction.^5,6^

In routine clinical practice, a COPD diagnosis is often based on a single initial spirometry test, and none of the above COPD guidelines recommend repeat spirometry testing when making the diagnosis of COPD, or indicate that a different result may be found if spirometry is repeated.^1–4^ Interestingly, although long-term variability has been reported for FEV_1_ and FVC in healthy subjects^7–9^ and for patients with COPD,^10,11^ and their year-to-year variability is reported in ERS/ATS lung function guidelines to be ±15%,^12^ similar evidence for variability of FEV_1_/FVC is lacking.

The aim of this study was, therefore, in a sample of subjects at risk for COPD, to investigate the long-term stability of a diagnosis on the basis of a once-only measurement of post-bronchodilator FEV_1_/FVC ratio in primary care. We examined shifts in diagnostic category (i.e., shifts between ‘obstructed’ and ‘non-obstructed’ and vice versa) after 1 year and 2 years. We used routine spirometry data from subjects who had entered a respiratory health diagnostic and annual monitoring service offered by primary care diagnostic centres in the Netherlands.

## Results

### Study subjects

**Figure 1:**
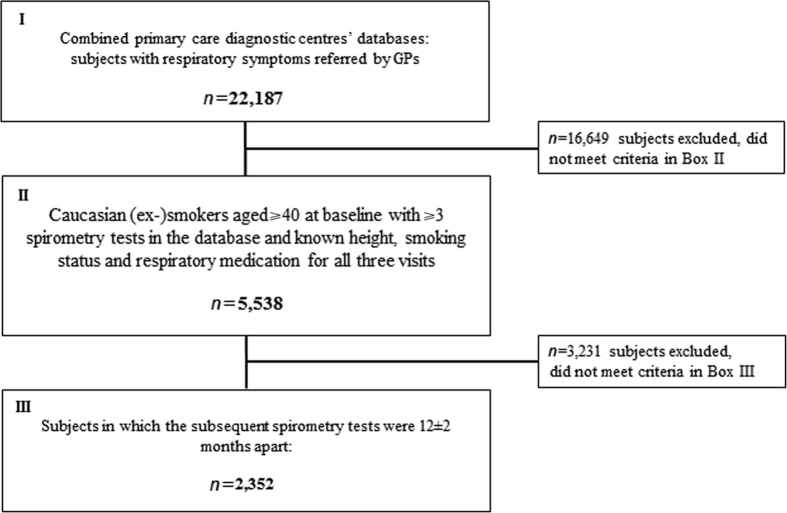
Selection of study subjects from the initial primary care diagnostic centres’ spirometry databases.

Of 22,187 subjects in the databases, 2,352 fulfilled the inclusion criteria (Figure 1). At baseline, airway obstruction was identified in 758 (32.2%) subjects by the LLN definition and in 1,097 (46.6%) subjects by the fixed FEV_1_/FVC definition. Table 1 shows baseline characteristics for obstructed and non-obstructed subjects according to the two definitions.

The average time between the initial diagnostic spirometry and the first follow-up measurement was 1.26 (s.d. 0.56) years, and 1.13 (s.d. 0.46) years between the first and second follow-up measurement.

### Agreement of obstruction status and FEV_1_/FVC values

**Figure 2:**
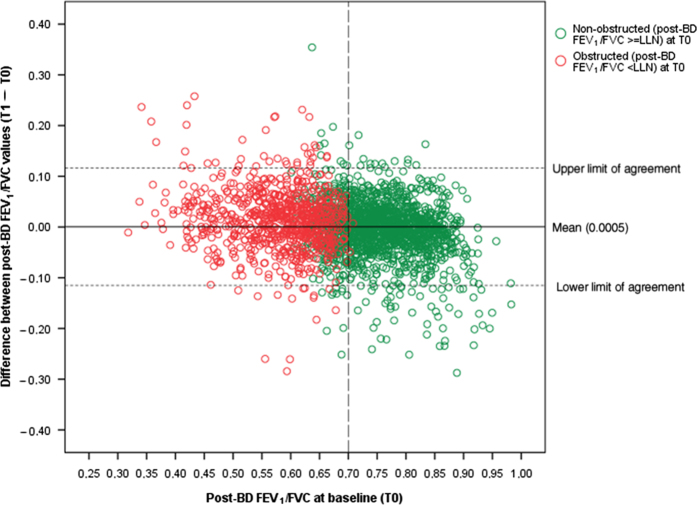
Difference between FEV_1_/FVC values measured at baseline (T0) and after 1 year (T1), plotted against T0. The coefficient of repeatability of the difference between the FEV_1_/FVC measurements at T0 and T1 was 0.115. BD, bronchodilator; FEV_1_, forced expiratory volume in 1 s; FVC, forced vital capacity; LLN, lower limit of normal.

Figure 2 shows the differences in post-BD FEV_1_/FVC between T0 and T1, plotted against the baseline (T0) value. The coefficient of repeatability of the within-subject difference between two consecutive FEV_1_/FVC measurements was 0.163 for the T0–T1 time interval, 0.157 for the T1–T2 time interval and 0.176 for the T0–T2 time interval.

**Figure 3:**
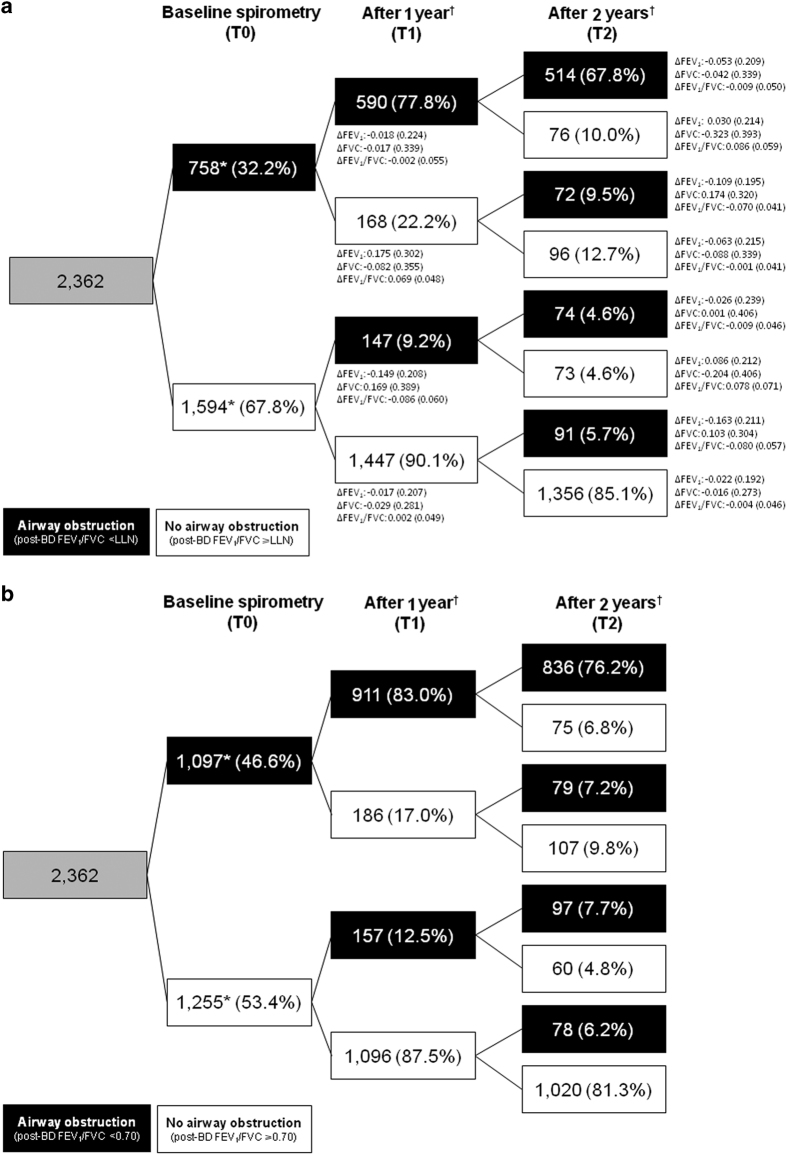
Change in obstruction status between baseline, year 1 and year 2 in respiratory symptomatic smokers and ex-smokers aged 40+ years. (**a**) Based on post-bronchodilator FEV_1_/FVC< or ⩾LLN. *Denominator for all proportions in the downstream cells. ^†^Indicates 12±2 months after previous test. BD, bronchodilator; FEV_1_, forced expiratory volume in 1 s (litres); FVC, forced vital capacity (litres); LLN, lower limit of normal. ∆FEV_1_, ∆FVC and ∆FEV_1_/FVC calculated as T1 minus T0, and T2 minus T1, respectively and reported as mean (s.d.). (**b**) Based on post-bronchodilator FEV_1_/FVC< or ⩾0.70.

Figure 3 shows the shifts between diagnostic categories by LLN (Figure 3a) and fixed ratio criterion (Figure 3b). According to the LLN criterion, 77.8% of subjects categorised as obstructed at baseline were still categorised as having airway obstruction after 1 year, and after 2 years only 67.8% had obstruction (Figure 3a). Figure 3a also shows mean (s.d.) changes in FEV_1_, FVC and FEV_1_/FVC between T0 and T1 and between T1 and T2 in the respective categories. Of the subjects without baseline airway obstruction (*n*=1,594), 90.1% remained unobstructed after 1 year and 85.1% after 2 years. Agreement between obstruction/non-obstruction status was ‘substantial’ when comparing T0 with T1 (Kappa=0.691, 95% confidence interval (95% CI)=0.660–0.722) and T0 with T2 (Kappa=0.671, 95% CI=0.640–0.702) classifications.

According to the fixed FEV_1_/FVC definition, 83.0% of initially obstructed subjects were still categorised as having airway obstruction after 1 year, and after 2 years only 76.2% had obstruction (Figure 3b). Of the subjects without baseline airway obstruction (*n*=1,255), 87.5% remained unobstructed after 1 year and 81.3% after 2 years. Again, agreement between obstruction/non-obstruction status was ‘substantial’ when comparing T0 with T1 (Kappa=0.707, 95% CI=0.678–0.736) and T0 with T2 (Kappa=0.695, 95% CI=0.666–0.724) classifications.

**Figure 4:**
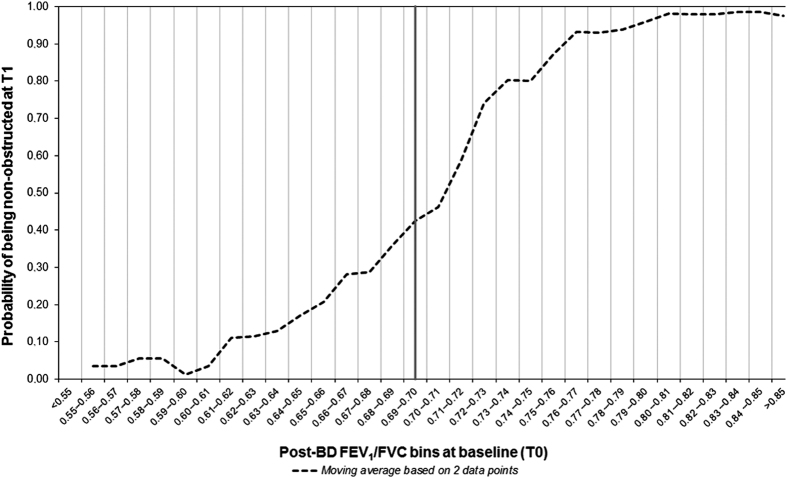
Probability of being non-obstructed after 1 year (T1) in relation to a subject’s post-BD FEV_1_/FVC at baseline T0. The graph shows moving averages based on two consecutive data points (i.e., values for the probabilities in the actual FEV_1_/FVC bin and the next bin) to ‘smooth’ the curve. BD, bronchodilator; FEV_1_, forced expiratory volume in 1 s; FVC, forced vital capacity.

Figure 4 shows that diagnostic shifts were observed across the full range of baseline FEV_1_/FVC values. Numbers (%) of patients with borderline results in terms of the fixed FEV_1_/FVC definition (e.g., FEV_1_/FVC between 0.68 and 0.72) were 337 (14.3%) at T0, 319 (13.6%) at T1 and 321 (13.6%) at T2. Of the 315 patients who shifted category in either direction between T0 and T1, only 65 (20.6%) were in the 0.68–0.72 range at T0.

### Factors associated with shifting between diagnostic categories

Several factors were independently associated with shifting from being obstructed at T0 to being non-obstructed at T1 when applying the LLN FEV_1_/FVC criterion. Higher body mass index (BMI) and baseline short-acting bronchodilator use increased the odds of shifting to non-obstructed (Table 2). Older age, baseline post-BD FEV_1_ <50% predicted, baseline inhaled corticosteroid use, and being a current smoker or using a short-acting bronchodilator at T1 reduced this odds.

Being male, older age, lower baseline post-BD FEV_1_% predicted, and being current smoker at baseline increased the odds of shifting from being non-obstructed to being obstructed at T1, whereas higher baseline BMI reduced these odds (Table 2).

Bronchodilator reversibility was not significantly associated with diagnostic shift in either direction.

## Discussion

### Main Findings

In primary care, establishing the presence or absence of airway obstruction when diagnosing COPD is often based on a single spirometry test. This is consistent with clinical guidelines for the diagnosis of COPD, which do not suggest that spirometry should be repeated to confirm the diagnosis. However, the short-term and long-term stability of a decreased post-bronchodilator FEV_1_/FVC ratio has not been reported. We investigated the shifts between diagnostic categories after an initial spirometry test in subjects for whom guidelines recommend investigation for COPD (age ⩾40, current or ex-smoker, with respiratory symptoms) and who were referred to a diagnostic service by their general practitioner (GP). We found that, depending on the definition of airway obstruction applied, after 1 year, 17–22% of subjects with airway obstruction at baseline were no longer categorised as such, and after 2 years, 24–32%; this shift was observed across a wide range of baseline FEV_1_/FVC values. Of subjects without airway obstruction at baseline, 10–13% were no longer non-obstructed after 1 year, and 15–19% after 2 years. With Kappa values in the range of 0.67–0.71 when comparing obstruction/non-obstruction status at baseline and after 1 and 2 years, respectively, agreement at a population level would be considered ‘substantial’, but the implications for individual patients may be important. Gender, age, BMI, post-BD FEV_1_% predicted, smoking status, and use of short-acting bronchodilators and inhaled corticosteroids were associated with shifts between diagnostic categories in the logistic regression models, but bronchodilator reversibility was not.

### Interpretation of findings in relation to previously published work

It is well accepted that FEV_1_ and FVC vary over time in both healthy persons and those with airways disease, with year-to-year variability reported as ±15% in the 2005 ATS/ERS guidelines for lung function testing,^12^ and that bronchodilator reversibility in patients with COPD varies when measured at 4-weekly intervals.^13^ However, the extent to which the FEV_1_/FVC ratio itself varies does not appear to have been documented. In a secondary analysis of the Lung Health Study dataset, which consists of 5-year follow-up data from 5,321 current smokers aged 35–60 years with mild-to-moderate obstructive pulmonary disease (defined at the time as baseline FEV_1_/FVC ratio ⩽0.75 and FEV_1_ 50–90% predicted),^14^ Akkermans *et al*.^15^ observed that classification as obstructed/non-obstructed was inconsistent for 24% of Lung Health Study participants between the initial screening and the first follow-up spirometry at 1 year. In another study examining the relationship between baseline obstruction and lung function decline from the present database, we noted that 36% of participants were excluded as they had changed obstruction category during an average of 3.4 years follow-up.^16^ However, we have not been able to trace any other studies that have reported the short-term or long-term consistency of a spirometric diagnosis of airway obstruction in subjects with COPD-like symptoms.

### Factors associated with diagnostic shift

Several factors were associated with shifting from obstructed to non-obstructed over 1 year. Age was a significant predictor of shift (in either direction), even with the age-adjusted LLN criterion. Other significant predictors included male gender, lower baseline FEV_1_% predicted and current smoking, all known predictors for the development of COPD.^17^ Higher baseline BMI was significantly associated with shifts in both directions—increasing the probability of shifting from obstructed to non-obstructed, and reducing the probability of shifting from non-obstructed to obstructed. Baseline bronchodilator reversibility (which might indicate greater underlying variability in lung function consistent with asthma) was not associated with diagnostic shift in either direction. Only limited information was available about medications, but use of inhaled corticosteroids at baseline appeared to reduce the probability of shifting from obstructed to non-obstructed.

### Strengths and limitations of this study

Particular strengths of our study are the large sample of subjects from primary care for whom guidelines recommend investigation for COPD (respiratory symptoms, age ⩾40, smoker/ex-smoker), the fact that all spirometry tests were performed by certified technicians using standardised protocols and equipment,^18^ that both pre- and post-bronchodilator spirometry were performed, and the existence of regional primary care programmes for ongoing monitoring of lung function as a part of routine patient care.

The study has some limitations as well. First, we assume that the patients being seen longitudinally in the primary care diagnostic centres are representative of a larger population, but we have no information on the precise reasons why some patients were scheduled for annual follow-up visits, whereas other patients were not. Selection may have occurred, as patients in the monitoring service may have been patients of special concern to their GPs; therefore, we cannot exclude the possibility that the number of variable spirometric findings was falsely elevated because of this. Also, patients with severe and unchanging disease may have been referred to secondary care medical specialists and have been lost to the primary care diagnostic centre monitoring service and, consequently, to our dataset. This might have caused a bias away from seeing consistent findings from year to year. Another limitation of the study is the fact that, because these follow-up visits are scheduled annually, we were not able to look at the variability of FEV_1_/FVC over shorter periods of time—for instance, within-day or week-to-week—as already reported for FEV_1_ and FVC. Further research is needed to establish the optimal interval between the initial spirometry test and a ‘verification test’ after some weeks or months. Clearly, regression to the mean effects could have a role in explaining our observations as, by chance alone, subjects with more extreme FEV_1_/FVC values are likely to show less extreme values at reassessment; furthermore, as the diagnosis of COPD is currently based on a specific FEV_1_/FVC value (whether <LLN or ⩽0.70), trivial changes could lead to a diagnostic shift for subjects with a baseline ratio just below or just above the relevant cut-off point. However, only about 20% of patients with a diagnostic shift had been in the borderline range of 0.68–0.72 at baseline, and diagnostic shifts were observed across the full range of baseline FEV_1_/FVC values (Figure 4). Finally, subjects non-obstructed at baseline may have been less likely to be enrolled in a diagnostic centre’s monitoring service and may therefore be under-represented in our dataset.

In clinical practice, a COPD diagnosis is often based on a single spirometry test. This is consistent with current guidelines, which recommend that smoking or ex-smoking subjects aged ⩾40 years with respiratory symptoms should be investigated for COPD and that the diagnosis should be ‘based on spirometry’, with no indication that it should be repeated to confirm the diagnosis. Given the importance of the FEV_1_/FVC ratio in making the diagnosis of COPD, and the known year-to-year variability of FEV_1_ and FVC of ±15%, we found it surprising that the variability of the ratio does not appear to have been reported previously. The current study shows that the FEV_1_/FVC ratio varies significantly over 1- and 2-year periods in subjects at a risk for COPD. Depending on the criterion for obstruction that is applied, one-off spirometry may lead to over- or under-diagnosis of COPD, and either of these may have a significant emotional impact on the patient.^19^ Further, diagnostic inaccuracy will almost certainly lead to over-treatment of some patients, with increased healthcare costs, increased risk for adverse effects and delay in identifying other treatable causes of respiratory symptoms, whereas other patients may be under-treated for COPD, contributing to unnecessary burden of disease. Clinical COPD guidelines should take this into account and recommend repetition of spirometry to verify the presence or absence of airway obstruction. An alternative view that has been increasingly heard in recent years is that the diagnosis of a heterogeneous multi-system condition such as COPD should not be based on a single number.^6,20,21^ This is especially relevant for primary care, where the vast majority of patients with early and mild COPD are diagnosed and treated.

### Conclusions

Although overall agreement between baseline and repeated diagnostic classification of airway obstruction may be technically classified as ‘substantial’ at a population level, a key finding of the present analysis was that up to one-third of people at risk for COPD who were found to have airway obstruction when referred for diagnostic spirometry by their GP had shifted to non-obstructed when routinely re-tested after 1 or 2 years. Similar shifts were seen with LLN and fixed-ratio criteria. Gender, age, BMI, baseline FEV_1_% predicted, smoking status and use of respiratory medication were associated with the probability of change in diagnostic category, but broncho-dilator reversibility was not. Given the implications described above for patients and the healthcare system, we do not believe that the diagnosis of COPD should be based on a single spirometry test.

## Materials and Methods

### Study setting and measurements

This observational study was based on all available spirometry tests from the period October 2001 to March 2010 from three regional primary care diagnostic centres (i.e., General Practice Laboratory Foundation Etten-Leur/Breda (SHL); Diagnostic Center Eindhoven (DC4U) and General Practice Laboratory East (SHO)) in the Netherlands. These diagnostic centres have offered a range of diagnostic tests, including spirometry, and other health services to hundreds of GPs in the south-western and south-eastern parts of the country since the mid- or late 1990s. When a subject consults his/her GP with respiratory symptoms and the GP suspects an underlying chronic respiratory condition (e.g., COPD or asthma), the subject can be referred to the diagnostic centre for pre- and post-bronchodilator spirometry testing. When a chronic respiratory condition is diagnosed or still suspected, the GP will usually enrol the subject in the diagnostic centre’s routine monitoring service for periodic (usually yearly) reassessment without further clinical selection.

As previously reported,^18^ all spirometry tests in the primary care diagnostic centres are performed by certified lung function technicians using personal computer-based digital volume sensor spirometers (SpiroPerfect; Welch Allyn, Delft, The Netherlands) and standardised calibration and measurement procedures.^18^ Subjects are instructed to withhold all bronchodilators before spirometry. The spirometry test results and accompanying demographic (gender, age), anthropometric (height, weight) and medical history information (including self-reported smoking status and history, respiratory symptoms, respiratory medications and exacerbations) are recorded during each visit using a standardised electronic format. Every spirometry test is assessed by a respiratory consultant and his/her interpretation and—if applicable—diagnostic advice is sent to the GP, together with the actual test results. Further details about the spirometry tests performed in the diagnostic centres are described elsewhere.^18^ At the time, the diagnostic centres did not electronically store spirometry test quality assessments in their databases. As only routine lung function and respiratory medical history data were used for our analyses and the investigators had no access to information on subjects’ identity or their medical records, no written informed consent was obtained.

### Subject selection and definitions for airway obstruction

For the current study, we selected subjects from the combined primary care diagnostic centres’ databases (*n*=22,187)^16^ who had risk factors for COPD and had complete questionnaire data and follow-up spirometry. The inclusion criteria were the following: being Caucasian; current or former smoker; aged ⩾40 years; complete data regarding height, history of cigarette smoking and respiratory medication use; and three or more post-bronchodilator spirometry tests available with 12±2 months (10–14 months) between tests.

We used post-bronchodilator FEV_1_/FVC values to classify the subjects as having or not having airway obstruction. The following two definitions of airway obstruction were applied:

- LLN cut-off (primary definition): post-bronchodilator FEV_1_/FVC below the subject’s age-specific lower limit of normal (LLN) value.^22^ Airway obstruction was classified as being present when the resulting standard deviation (s.d.) score (also known as ‘standardised z-score’) was <−1.645. This corresponds with the fifth percentile.
- Fixed cut-off point (secondary definition): post-bronchodilator FEV_1_/FVC <0.70. This is the criterion for airway obstruction that is still recommended in clinical COPD guidelines.^1–4^

Global Lung Initiative prediction equations^23^ were used to calculate the LLN values for FEV_1_/FVC and %predicted values for FEV_1_.

### Analysis

Subjects were categorised as showing airway obstruction or not at their consecutive measurements (baseline (T0), 1 year (T1) and 2 years (T2)). The Kappa statistic and its 95% CI were calculated to express the level of agreement between T0 and T1 and between T0 and T2 diagnostic status, respectively. The following classification in terms of strength of agreement for the kappa coefficient was used: Kappa ⩽0=poor agreement; 0.01 to 0.20=slight; 0.21 to 0.40=fair; 0.41 to 0.60=moderate; 0.61 to 0.80=substantial; and 0.81 to 1=almost perfect.^24^ A modified Bland–Altman^25^ plot was generated to graphically express differences in FEV_1_/FVC between T0 and T1, compared with the baseline (T0) value, and the coefficient of repeatability^26^ was calculated to express the within-subject repeatability (or absolute reliability) of the two consecutive FEV_1_/FVC measurements. The coefficient of repeatability is the value below which the absolute differences between two measurements would lie with 0.95 probability. Both random and systematic errors are taken into account in the coefficient.^26^

In univariate analyses we calculated the probability of being (non-)obstructed after 1 year in relation to a subject’s baseline post-bronchodilator FEV_1_/FVC value. We also explored subject characteristics that predicted shifting diagnostic category between T0 and T1 measurements for the primary (LLN) definition of obstruction using multivariable logistic regression models. Covariates in these analyses were gender, age (at T0), BMI (at T0), severity of airway obstruction (categorised according to GOLD as mild, moderate and (very) severe obstruction, based on % predicted FEV_1_, at T0),^1^ reversibility of obstruction (yes/no ∆FEV_1_ ⩾200 ml and ⩾12%, at T0),^27^ smoking status (current smoker yes/no, at T0 and T1), use of short-acting bronchodilators (yes/no, at T0 and T1), use of long-acting bronchodilators (yes/no, at T0 and T1) and use of inhaled corticosteroids (yes/no, at T0 and T1). The changes in smoking status, short-acting bronchodilator use, long-acting bronchodilator use, and inhaled corticosteroid use were expressed in the models using interaction terms of the respective T0 and T1 covariates, but dropped at a later stage as they were not statistically significant. Separate models were constructed for each of the two possible directions of shifting (i.e., from obstructed to non-obstructed or vice versa). Associations were expressed as odds ratios with 95% CIs. Tests were two-sided. *P*<0.10 was considered statistically significant. IBM SPSS Statistics Version 22 (Armonk, NY, USA) was used for the analyses.

## Funding

The extraction of data from the primary care diagnostic centre's databases was supported by a research grant from Boehringer Ingelheim, the Netherlands.

## Figures and Tables

**Table 1:** Baseline (T0) characteristics of the study sample (*n*=2,352) for the two definitions of airway obstruction

	*LLN definition*		*Fixed definition*	
	*Airway obstruction*^a^ (n*=758)*	*No airway obstruction*^a^ (n*=1,594)*	*Airway obstruction*^b^ (n*=1,097)*	*No airway obstruction*^b^ (n*=1,255)*
*Demographics*				
Age (years)	61.0 (10.2)	60.0 (10.8)	63.2 (10.2)	57.8 (10.3)
Males, *n* (%)	473 (62.4)	794 (49.8)	703 (64.1)	564 (44.9)
BMI (kg/m^2^)	26.1 (4.4)	27.7 (5.0)	26.4 (4.3)	28.0 (5.2)
Current smokers, *n* (%)	413 (54.5)^c^	649 (40.7)^d^	552 (50.3)	510 (40.6)
				
*Lung function*				
FEV_1_ post-BD (L)	2.02 (0.64)	2.47 (0.71)	2.07 (0.64)	2.55 (0.70)
% Predicted^a^	64.9 (13.5)	82.0 (14.3)	67.9 (14.2)	84.0 (13.9)
⩾80%, *n* (%)^e^	103 (13.6)	878 (55.1)	223 (20.3)	758 (60.4)
50 to <80%, *n* (%)^e^	544 (71.8)	697 (43.7)	751 (68.5)	490 (39.0)
30 to <50%, *n* (%)^e^	107 (14.1)	18 (1.1)	118 (10.8)	7 (0.6)
<30%, *n* (%)^e^	4 (0.5)	1 (0.1)	5 (0.5)	0
FEV_1_ post-BD minus pre-BD, % (s.d.)	11.7 (11.6)	8.1 (8.4)	11.2 (11.1)	7.5 (7.9)
Reversible^f^, *n* (%)^e^	271 (35.8)	380 (23.8)	371 (33.8)	280 (22.3)
FVC post-BD (L)	3.49 (0.98)	3.27 (0.92)	3.41 (0.97)	3.27 (0.92)
% Predicted^a^	87.3 (14.8)	84.6 (13.9)	86.7 (14.9)	84.4 (13.6)
FEV_1_/FVC post-BD	0.58 (0.08)	0.76 (0.07)	0.61 (0.08)	0.78 (0.05)
				
*Respiratory medication*				
Any respiratory medication, *n* (%)	542 (71.5)	1216 (76.3)	771 (70.3)	987 (78.6)
Short-acting bronchodilator, *n* (%)	307 (40.5)	623 (39.1)	421 (38.4)	509 (40.6)
Long-acting bronchodilator, *n* (%)	296 (39.1)	586 (36.8)	416 (37.9)	466 (37.1)
Inhaled corticosteroid, *n* (%)	360 (47.5)	908 (57.0)	512 (46.7)	756 (60.2)
				
*Exacerbations*^g^ *and respiratory symptoms*^h^				
Exacerbation(s) in past year, *n* (%)	301 (39.7)	633 (39.7)	431 (39.3)	503 (40.1)
Chronic cough, *n* (%)	474 (62.5)	800 (50.2)	637 (58.1)	639 (50.9)
Chronic sputum, *n* (%)	518 (68.3)	958 (60.1)	742 (67.6)	733 (58.4)
Daytime dyspnoea, *n* (%)	550 (72.5)	1097 (68.8)	769 (70.1)	876 (69.8)
Night-time dyspnoea, *n* (%)	129 (17.0)	330 (20.7)	189 (17.2)	272 (21.7)
Allergic symptoms, *n* (%)	236 (31.1)	666 (41.8)	331 (30.2)	572 (45.6)

Abbreviations: BD, bronchodilator; BMI, body mass index; FEV_1_, forced expiratory volume in 1 s; FVC, forced vital capacity; GLI, Global Lung Initiative; GOLD: Global Initiative for Chronic Obstructive Lung Disease; LLN, lower limit of normal.

a Based on GLI reference equations.^23^

b Based on post-bronchodilator FEV_1_/FVC <0.70.

c Smoking status at T1: in smokers at baseline—83% still current smoker, 17% former smoker; in former smokers at baseline: 88% still former smoker, 12% current smoker.

d Smoking status at T1: in smokers at baseline—85% still current smoker, 15% former smoker; in former smokers at baseline: 94% still former smoker, 6% current smoker.

e Grouping of FEV1% predicted based on GOLD classification of severity of airway obstruction.^1^

f Reversibility: FEV_1_ ⩾12% and ⩾200 ml 15 min after 400 μg salbutamol administered by spacer.^27^

g Exacerbations defined as one or more self-reported episodes with aggravated respiratory symptoms lasting for several days in the past year.

h Data on exacerbations and/or respiratory symptoms were missing in 676 of the 2,352 subjects (29%); therefore, these numbers and percentages are based on 1,676 subjects.

**Table 2:** Results from multivariable logistic regression models looking at factors associated with diagnostic shift between baseline (T0) and 1-year measurements (T1) using post-BD lower limit of normal (LLN) FEV_1_/FVC cut-off points to define the presence or absence of airway obstruction

*Determinant*	*Shift from obstructed at baseline to non-obstructed after 1 year*				*Shift from non-obstructed at baseline to obstructed after 1 year*			
	*OR*	*95% CI*		P	*OR*	*95% CI*		P
Males (versus females)	0.81	0.55	1.20	0.288	**1.41**	**0.96**	**2.05**	**0.077**
Age (per year older)	**0.98**	**0.96**	**1.00**	**0.069**	**1.03**	**1.01**	**1.04**	**0.006**
BMI (per kg/m^2^ higher)	**1.07**	**1.03**	**1.12**	**0.002**	**0.93**	**0.89**	**0.97**	**<0.001**
Significant bronchodilator reversibility^a^ (versus no significant reversibility)	0.76	0.50	1.13	0.178	1.31	0.87	1.98	0.192
Post-BD FEV_1_ 50 to <80% predicted (versus ⩾80% predicted)	0.66	0.39	1.10	0.113	**2.94**	**1.96**	**4.39**	**<0.001**
Post-BD FEV_1_ <50% predicted (versus ⩾80% predicted)	**0.41**	**0.19**	**0.87**	**0.020**	**6.80**	**2.19**	**21.08**	**0.001**
Current smoker at baseline^b^ (versus former smokers)	1.12	0.62	2.02	0.694	**3.43**	**1.53**	**7.66**	**0.003**
Current smoker after 1 year^b^ (versus former smokers)	**0.43**	**0.24**	**0.78**	**0.005**	0.65	0.29	1.45	0.294
Short-acting bronchodilator^c^ at baseline^b^ (versus no short-acting BD)	**1.58**	**0.95**	**2.61**	**0.076**	1.38	0.84	2.27	0.207
Short-acting bronchodilator^c^ after 1 year^b^ (versus no short-acting BD)	**0.61**	**0.37**	**1.01**	**0.053**	0.74	0.45	1.21	0.229
Long-acting bronchodilator^b^^,^^d^ (versus no long-acting BD)	1.20	0.59	2.42	0.614	0.89	0.43	1.84	0.761
Long-acting bronchodilator after 1 year^b^^,^^d^ (versus no long-acting BD)	0.75	0.38	1.47	0.397	1.26	0.61	2.61	0.527
Inhaled corticosteroids at baseline^b^ (versus no ICS)	**0.56**	**0.30**	**1.05**	**0.069**	0.78	0.48	1.26	0.312
Inhaled corticosteroids after 1 year^b^ (versus no ICS)	1.39	0.76	2.53	0.284	0.98	0.60	1.60	0.940

Odds ratios that are statistically significantly different from 1 are printed in bold.

Abbreviations: BD, bronchodilator; BMI, body mass index; CI, confidence interval; FEV1, forced expiratory volume in 1 s; FVC, forced vital capacity; ICS, inhaled corticosteroids; LLN, lower limit of normal; OR, odds ratio.

a Significant bronchodilator reversibility: increase in FEV_1_ ⩾12% and ⩾200 ml 15 min after 400 μg salbutamol administered by spacer.^27^

b Interaction terms for T0 and T1 values of smoking status, short-acting BD use, long-acting BD use and ICS use were not statistically significant.

c Short-acting β_2_-agonists and/or anticholinergics.

d Long-acting β_2_-agonists and/or anticholinergics.

## Notes

TRS is an Associate Editor of *npj Primary Care Respiratory Medicine*, but was not involved in the editorial review of, nor the decision to publish this article. The other authors declare no conflict of interest.
